# Pediatric lymphomas: overview and diagnostic challenges

**DOI:** 10.1007/s00428-024-03980-9

**Published:** 2024-12-21

**Authors:** John Kim Choi, Leticia Quintanilla-Martinez

**Affiliations:** 1 Department of Pathology, The University of Alabama at Birmingham, WP P30N, 619 19Th Street South, Birmingham, AL 35249-7331 USA; 2 Institute of Pathology and Neuropathology, Eberhard Karls University of Tuebingen and Comprehensive Cancer Center, University Hospital Tuebingen, Liebermeisterstr. 8, 72076 Tuebingen, Germany

**Keywords:** Pediatric lymphomas, *IRF4* rearrangement, 11q alterations, EBV + lymphoproliferations, Pediatric-type follicular lymphoma, Burkitt lymphoma, Hodgkin lymphoma

## Abstract

Only 10% of new lymphoma diagnoses in the USA occur in children < 15 years. Although the same diagnostic criteria apply to both adult and pediatric lymphomas, there are important differences in some lymphoma subtypes. These differences are recognized by the World Health Organization (WHO) with the recent 2022 classification of pediatric tumors including pediatric hematopoietic tumors. Here, we review the WHO classification scheme for pediatric lymphomas and summarize the diagnostic criteria, recent genetic findings, and differences from their adult counterparts for some subtypes including those yet to be included as a definitive subtype. In general, there are differences in relatively frequency, genetic mutation, and prognosis with the pediatric counterpart often having better prognosis. Emerging B-cell lymphomas with recurrent gene alterations such as *IRF4* rearrangement and 11q gain/loss chromosomal alterations will be reviewed. The overlapping pathological, clinical, and molecular features between pediatric-type follicular lymphoma (PTFL) and pediatric nodal marginal zone lymphoma (PNMZL) suggesting one disease with broad morphological spectrum will be discussed. The pathogenetic role of EBV in subclassifying Burkitt lymphoma is highlighted. The revised classification of the EBV-positive lymphoproliferative disorders in children is discussed. This review will focus on novel findings, areas of special interest, and diagnostic challenges in pediatric lymphomas.

## Introduction

Approximately 80,800 new diagnoses of lymphomas, including 72,000 non-Hodgkin lymphomas (NHL) and 8800 of Hodgkin lymphomas (HL) occur in the USA yearly, representing < 1% of all new US cancer. Of these, approximately 970 (620 NHL and 350 HL) occur in children < 15 years [1]. Hence, most pathology articles, reviews, and books focus on adult lymphoma with the pediatric counterpart often only briefly or not mentioned at all within the context of the more numerous adult lymphomas. However, there are important differences, most commonly within frequencies, molecular findings, and prognosis [2–12]. The recent 2022 World Health Organization (WHO) classification of pediatric tumors included a section on pediatric hematopoietic tumors (Pediatric WHO) [13]. The 2024 WHO 5th edition and the 2022 International Consensus Classification (ICC) on hematopoietic neoplasm [14, 15] focus mainly on adults; although, some tumors prevalent in children are detailed and some sections highlight the differences between adults and pediatric lymphomas.

This review gives an overview of the WHO classification scheme of pediatric lymphoma, focusing on diagnostic criteria, recent genetic findings, and some differences from their adult counterparts. The common definitive lymphoma subtypes of Pediatric WHO and some uncommon subtypes (both definitive and those yet to be included in the Pediatric WHO) are reviewed (see Tables 1 and 2). Some uncommon lymphoma subtypes and most cutaneous lymphomas, including mycosis fungoides, will not be reviewed. In general, the diagnostic criteria for pediatric lymphoma subtypes are like their adult counterparts, but the encountered distribution of subtypes is different. For example, follicular lymphoma (FL) is common in adults but rare in children and exceptionally rare, if limited to those with *IGH::BCL2*. Other differences include genetic differences and prognosis. For example, pediatric classic Hodgkin lymphoma (CHL) has higher mutational burden and better prognosis than its adult counterpart. The review, unless otherwise states, summarizes findings in children and list some differences from their adult counterparts.

**Table 1:** Relative frequency of pediatric B-cell lymphomas

Common
Burkitt lymphoma
Diffuse large B-cell lymphoma, NOS
Less common and rare
B-lymphoblastic lymphoma
Primary mediastinal B-cell lymphoma
High-grade B-cell lymphoma with 11q aberrations
Large B-cell lymphoma with *IRF4* rearrangement
Mediastinal gray-zone lymphoma
Pediatric-type follicular lymphoma
Pediatric nodal marginal zone lymphoma
High-grade B-cell lymphoma, NOS
High-grade “double-hit” B-cell lymphoma
T-cell/histiocyte-rich large B-cell lymphoma
Very rare
EBV + DLBCL, NOS
ALK + large B-cell lymphoma
Lymphomatoid granulomatosis
Plasmablastic lymphoma
Extranodal marginal zone lymphoma
Primary CNS lymphoma
Primary effusion lymphoma

Modified from [9, 13], Underlined lymphoma are definitive subtypes in WHO Pediatric Haematolymphoid disorders

*NOS* not otherwise specified, *EBV* Epstein-Barr virus, *DLBCL* diffuse large B-cell lymphoma, *ALK* anaplastic lymphoma kinase

Common, less common and rare, and very rare are terminology used by COG [9] but not defined. Common is rougly > 10% while very rare is < 1–2%

**Table 2:** Pediatric T-cell lymphomas and relative frequency

Common
T-lymphoblastic lymphoma
Anaplastic large-cell lymphoma, ALK positive
Less common and rare
Subcutaneous panniculitis-like T-cell lymphoma
Hepatosplenic T-cell lymphoma
Mycosis fungoides
Primary cutaneous CD30 + lymphoproliferative disorders
Lymphomatoid papulosis
Primary cutaneous anaplastic large-cell lymphoma
Peripheral T-cell lymphoma, NOS
Anaplastic large-cell lymphoma, ALK negative
Extranodal NK/T-cell lymphoma
Primary cutaneous gamma-delta T-cell lymphoma
Very rare
EBV-positive lymphoproliferations of childhood
Severe mosquito bite allergy
Systemic chronic active EBV disease, systemic
Systemic EBV + T-cell lymphoma of childhood
Hydroa vacciniforme lymphoproliferative disorder
Aggressive NK-cell leukemia
Primary cutaneous CD4 + small/medium T-cell LPD
T-cell large granular lymphocytic leukemia
Enteropathy-associated T-cell lymphoma
Adult T-cell leukemia/lymphoma

Modified from [9, 13], underlined lymphoma are definitive subtypes in WHO Paediatric WHO Haematolymphoid disorders

*LPD* lymphoproliferative disorder, *EBV* Epstein-Barr virus, *NOS* not otherwise specified, *NK* natural killer cell, *ALK* anaplastic lymphoma kinase

Common, less common and rare, and very rare are terminology used by COG [9] but not defined. Common is rougly > 10% while very rare is < 1–2%

### Precursor B-cell lymphomas

#### B-lymphoblastic lymphoma (B-LBL)

B-LBL is a neoplasm of B lymphoblasts that present as an extramedullary mass without significant bone marrow (BM) or peripheral blood (PB) involvement, arbitrary defined as < 25% blasts. Diagnostic criteria are based on the presence of blasts with B cell (CD19, CD20, CD22, PAX5, and/or CD79a) and immature (CD34, TdT, CD133, and/or NG2) immunophenotypic markers, ideally distinct from normal precursor B cells (hematogones). More commonly, precursor B-cell neoplasms present as leukemic (B-ALL) with ≥ 25% BM or PB blasts or ≥ 20% blasts without mass lesion. Molecular studies on B-ALL are identifying ever increasing genetic mutational subtypes with differing prognosis [16–18]. A study on the mutational landscape of B-LBL shows similar mutations to those in B-ALL, but genes associated with B-cell development, such as *PAX5* and *ETV6* are more common in B-ALL, while mutations in epigenetic regulators such as *ARID1A*, *EP300*, and *KMT2D* are more common in B-LBL [19]. In general, the outcome of B-LBL is excellent with 90% 5-year event-free survival (EFS) [20]. Unlike B-ALL genetic subtypes, the prognostic significance of genetic subtypes in pediatric B-LBL remains unclear.

### Mature B-cell lymphoma with medium to large B-cell or blastoid morphology

Pediatric B-cell NHLs with medium to large B-cell/blastoid morphology represent 60–70% of pediatric NHL with the most common being Burkitt lymphoma (BL, 30–40% of pediatric NHL), followed by diffuse large B-cell lymphoma, not otherwise specified (DLBLCL, NOS, 20–30% of pediatric NHL). The frequency of the latter will continue to decrease as more biologic subtypes are identified and given their own categories, such as large B-cell lymphoma with *IRF4* rearrangement (LBCL-*IRF4*) and high-grade/large B-cell lymphoma with 11q aberration (HG/LBCL-11q). The need for exact subtyping of lymphomas with large B-cell morphology, important in adults, is not as critical in the pediatric counterparts because they are treated similarly with excellent prognosis with 93.9% 3-year EFS [9]. With the exception of primary mediastinal large B-cell lymphoma (PMBL) [21], some have better outcomes like LBCL-*IRF4* suggesting potential de-escalation of therapy in the near future [22, 23]. The distinguishing features of these lymphomas are listed in Table 3.

**Table 3:** Characteristics of pediatric B-cell lymphoma with medium to large-cell/blastic morphology

B-cell lymphoma subtype	Pattern/site/	Immunophenotype	Genetics
Burkitt Lymphoma	Diffuse, follicular in early presentation Starry-sky pattern Often extranodal	EBER + subset GC phenotype, BCL2 − , LMO2 − , Ki67 + +	*t*(8;14)/*t*(2;8)/*t*(8;22) *MYC-*R
HGBCL-11q	BL-like or large cell cytology Starry-sky pattern with coarse apoptotic bodies. Mainly nodal disease	LMO2 + (50%), CD56 + subset, CD16 + subset	Array CGH, NGS, or FISH for 11q gain/loss 11q loss alone
DLBCL	Diffuse	Mostly GC, occasional non-GC	3q27 (minor subset) *BCL6-*R
Primary mediastinal LBCL	Diffuse, often fibrotic, mediastinum, regional lymph nodes, soft tissue	CD30dim, CD23 + , CD200 + , MAL1 + , PDL1 + , PDL2 +	*PDL1, PDL21L2:* amplification, rearrangement
LBCL-*IRF4*	Follicular, diffuse or follicular and diffuse/Waldeyer ring, cervical Lymph nodes, GI	CD5 + subset, BCL6 + , CD10 + (80%), MUM1 + +	*t*(6;14) *IRF4-*R (cryptic in 10%) *IRF4* mutations *TP53* mutations No *BCL6*-R in children
EBV + DLBCL, NOS*	THRLBCL pattern Mainly nodal disease	Mostly non-GC, CD30 + , CD10 − , MUM1 + , BCL6 ± , MEF2B + , PD1 + , PD-L1 + EBV latency II (mostly)	

*No evidence of immunodeficiency

Modified from [13]

*HGBCL-11q* high-grade B-cell lymphoma with 11q aberrations, *DLBCL* diffuse large B-cell lymphoma, *LBCL* large B-cell lymphoma, *LBCL-IRF4* large B-cell lymphoma with *IRF4* rearrangement, *EBV* Epstein-Barr virus, *NOS* not otherwise specified, *GI* gastrointestinal tract, *GC* germinal center, *THRLBCL* T-cell/histiocyte rich large B-cell lymphoma, *R* rearrangement

#### Primary mediastinal large B-cell lymphoma (PMBL)

PMBL is a rare large B-cell lymphoma (1–2% of pediatric NHL) occurring in the anterior mediastinum with involvement of regional lymph nodes and/or distant soft tissue, and characterized by specific immunophenotype and molecular findings (Table 1). Diagnostic criteria are large B cells with at least partial expression of CD23 and CD30 and ideally positive for MAL and CD200. These are often CD30 + with some morphologic overlap with classic Hodgkin lymphoma (CHL) but can be distinguished by the expression of uniform CD45, CD19, CD79a, OCT2, and BOB1. PMBL can be distinguished from DLBCL by its characteristic immunophenotype, including expression of MAL, CD200, PDL1, PDL2 [24–26], molecular findings of *CIITA* abnormalities, and *PDL1*/*PDL2* gains [27]. Prognosis is good with 85.9% 3-year EFS similar to adult PMBL but inferior to other pediatric large cell lymphomas [9, 21, 28, 29].

#### Mediastinal gray-zone lymphoma (MGZL)

MGZL is an uncommon mature B-cell lymphoma (< 4% of pediatric NHL), first introduced in the 2008 WHO classification as “B-cell lymphoma, unclassifiable, with features intermediate between diffuse large B-cell lymphoma and classic Hodgkin lymphoma” and now designated MGZL. This subtype is localized to the mediastinum with conflicting morphologic and immunophenotypic features, i.e., morphology of CHL or PMBL, but the immunophenotype supporting the other disease [30, 31]. Composite areas of CHL and PMBL are not considered MGZL. New genetic data support the recognition of this disease as a discrete entity, most probably originating from thymic B cells [32, 33]. Cases outside the mediastinum should be classified as DLBCL, NOS. Rare EBV + MGZL have been reported [34], but most are best classified as EBV + DLBCL. This lymphoma predominates in males and typically presents in patients age 20–40 years with a small number reported in children [30, 35]. The prognosis in pediatric MGZL is unclear. However, it is well documented that adult MGZL has a much worse prognosis than pediatric PMBL [31]. Nevertheless, a similar comparison between pediatric MGZL and pediatric PMBL is not available in the literature.

#### Diffuse large B-cell lymphoma, not otherwise specified (DLBCL, NOS)

DLBCL, NOS is a large B-cell lymphoma that does not meet criteria for other well-defined large B-cell lymphoma subtypes. The vast majority, unlike the adult counterparts, are of germinal center B-cell (GCB) phenotypes (positive for CD10, BCL6); the rare non-GCB type has a similar prognosis [36]. The molecular changes and gene mutations are similar to those seen in adult DLBCL except for the rarity of *t*(14;18) translocation [37, 38].

#### EBV-positive diffuse largeB-cell lymphoma, not otherwise specified (EBV + DLBCL, NOS)

**Fig. 1:**
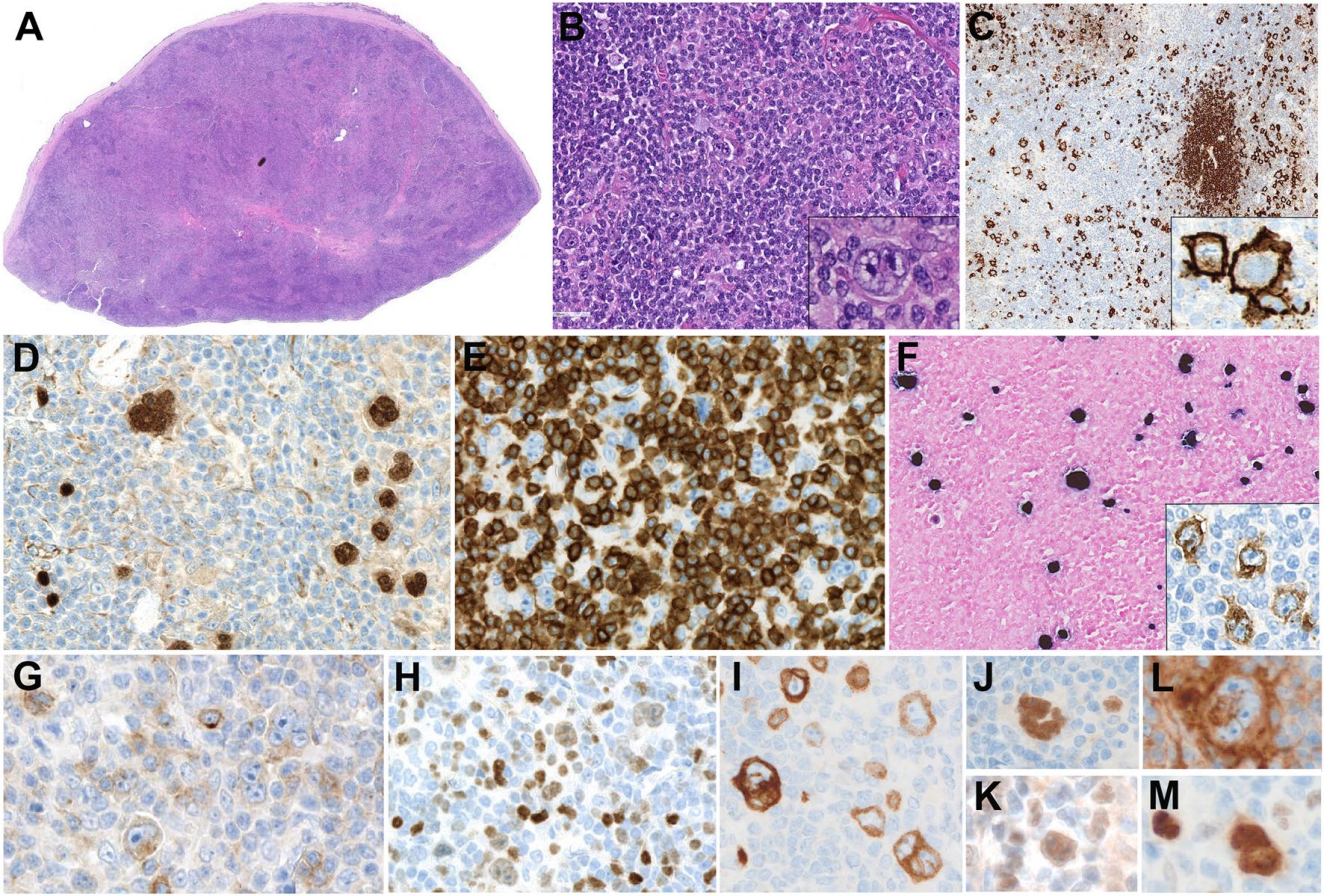
EBV + diffuse large B-cell lymphoma in a 16-year-old boy. **A** Panoramic view of a lymph node with H&E stain. Note the loss of normal architecture (snap-shot).** B** Higher magnification shows predominantly a small cell infiltrate with intermingled, scattered large atypical cells, some mimicking Reed-Sternberg cells. Inset: A large binucleated tumor cells with prominent basophilic nucleoli mimicking a Reed-Sternberg cell (original magnification 200 × , inset, 600 × ; hematoxylin and eosin). **C** CD20 stain shows residual small B-cell nodules and interfollicular scattered large cells. Inset: higher magnification shows the strong homogeneous CD20 positivity in the large atypical cells (original magnification 100 × , inset, 600 × ; immunostaining). **D** The large atypical cells are strongly PAX5 positive.** E** The large atypical cells are surrounded by small, reactive, CD3 + T cells. **F** EBER in situ hybridization is positive in large and small lymphocytes. Inset: The large cells are also LMP1 positive but remained EBNA2 negative (not shown). **G–H** Note that the atypical lymphoid cells are weakly positive for CD30 and MUM1, unlike classic Hodgkin lymphoma. **I** CD79a is positive in the atypical large cells. **J**, **K**, **L**, **M** The Reed-Sternberg-like cells are positive for MEF2B (**J**), BCL6 (**K**), PD-L1 (**L**), and OCT-2 (**M**) (**D**, **E**, **F**, **G**, **H**, **I**, **J**, **K**, **L**, **M** original magnification 400 × , immunostains)

EBV + DLBCL, NOS is a rare in children [39, 40] but more frequent (5% of NHL) in EBV endemic regions. The exact frequency may be higher because EBV studies are not usually performed on de-novo DLBCL [41]. Diagnostic criteria are DLBCL with EBV expression (> 80% EBER + tumor cells) and absence of underlying immunodeficiency (congenital or acquired). The morphology is variable but a T-cell/histiocyte-rich large B-cell pattern is frequently seen (Fig. 1A–M) [34, 42, 43]. The cytology of the tumor cells ranges from centroblasts, immunoblasts, or Hodgkin-Reed-Sternberg (HRS)-like cells embedded in a rich inflammatory background. The tumor cells show a B-cell phenotype with expression of the transcription factors BOB1 and OCT2 and often a non-GCB phenotype with lack of CD10 expression but expression of MUM1, BCL6, and MEF2B (Fig. 1J–K). Most cases are CD30 + , and a minority of cases express CD15, which raises the differential diagnosis with CHL (Fig. 1G–H). PDL1 and PDL2 are often expressed in young patients when compared with the elderly group (95% vs. 11%), suggesting a different pathogenetic mechanism involving immune escape rather than immunodeficiency (Fig. 1L). LMP1 is expressed in the majority of the cases but not EBNA2 [39, 41]. EBNA2 positivity requires exclusion of infectious mononucleosis or underlying immunodeficiency before a diagnosis of EBV + DLBCL with latency 3 can be rendered. The prognosis of pediatric EBV + DLBCL is good with 80% 5-year PFS [34, 42, 43] and better than that of the adult counterpart.

#### Large B-cell lymphoma with IRF4-rearrangement (LBCL-IRF4)

**Fig. 2:**
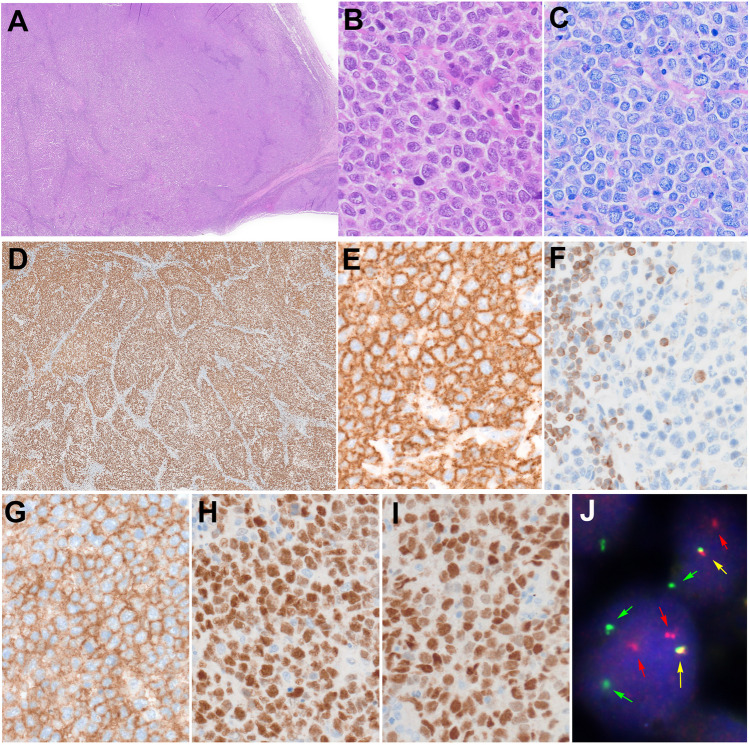
Large B-cell lymphoma with *IRF4* rearrangement. **A** Panoramic view of a lymph node with H&E stain. Note the vaguely nodular and diffuse growth pattern (original magnification 20 × ; hematoxylin and eosin). **B** Higher magnification shows medium-sized cells with open chromatin, inconspicuous nucleoli, and a rim of eosinophilic cytoplasm. Some mitoses are depicted. **C** Giemsa stain confirms the cytological features of the tumor cells (original magnification 400 × ; hematoxylin and eosin and Giemsa stains). **D** A panoramic view of the lymph node stained with MIB1. The stain reveals the predominantly nodular growth pattern of the neoplasia (original magnification 20 × ; immunostaining). **E** The tumor cells are CD20 positive. **F** BCL2 is negative in the tumor cells but positive in the reactive T cells. **G** The tumor cells are CD10, BCL6 (**H**), and IRF4/MUM1 (**I**) positive (**E**, **F**, **G**, **H**, **I** original magnification 400 × ; immunostainings). **J** FISH assay with break apart probes for *IRF4* demonstrates an *IRF4* break with one colocalized signal (yellow arrow) and one or two split signals (green and red arrows) consistent with gene rearrangement

LBCL-*IRF4* was introduced as a provisional entity in 2017 [44], now recognized as a definitive entity [14]. LBCL-*IRF4* is an uncommon large B-cell lymphoma (2–5% of pediatric NHL) [23, 45] with a follicular, diffuse, or follicular and diffuse pattern (Fig. 2). The disease occurs mainly in children and young adults (< 35 years) preferentially involving the Waldeyer’s ring, head, and neck lymph nodes (LN), and less frequently the gastrointestinal tract but can involve other sites [46]. Morphologically, it is composed predominantly of medium-sized blasts, but centroblastic cytology can be observed. The tumor cells express B-cell markers, CD10, BCL6, and IRF4/MUM1. CD5 expression is common in children. BCL2 is positive in half of the cases but it is not associated to *t*(14:18). IRF4/MUM1 positive DLBCL should be investigated for *IRF4* rearrangement. FISH analysis shows *IG::IRF4* in 90% of cases. When absent (usually cryptic translocation), the presence of IGH/K/L break in the absence of *BCL2, BCL6*, and *MYC* rearrangements and/or the presence of *IRF4* mutations support the diagnosis of LBCL-*IRF4*. Unlike the adult counterpart, pediatric LBCL-*IRF4* shows no *BCL6* rearrangements [46–48]. *BCL2* and *MYC* rearrangement should be absent. Molecular studies have revealed a GCB-type gene expression profile despite the strong expression of IRF4/MUM1 and frequent mutations in *IRF4* and NF-kB-related genes [43]. Prognosis is outstanding, with 100% 5-year EFS [23, 49] with a possibility of curative surgical resection in a localized disease [50]; *TP53* mutations are not rare but do not seem to alter the excellent prognosis [43].

#### Burkitt lymphoma (BL)

**Fig. 3:**
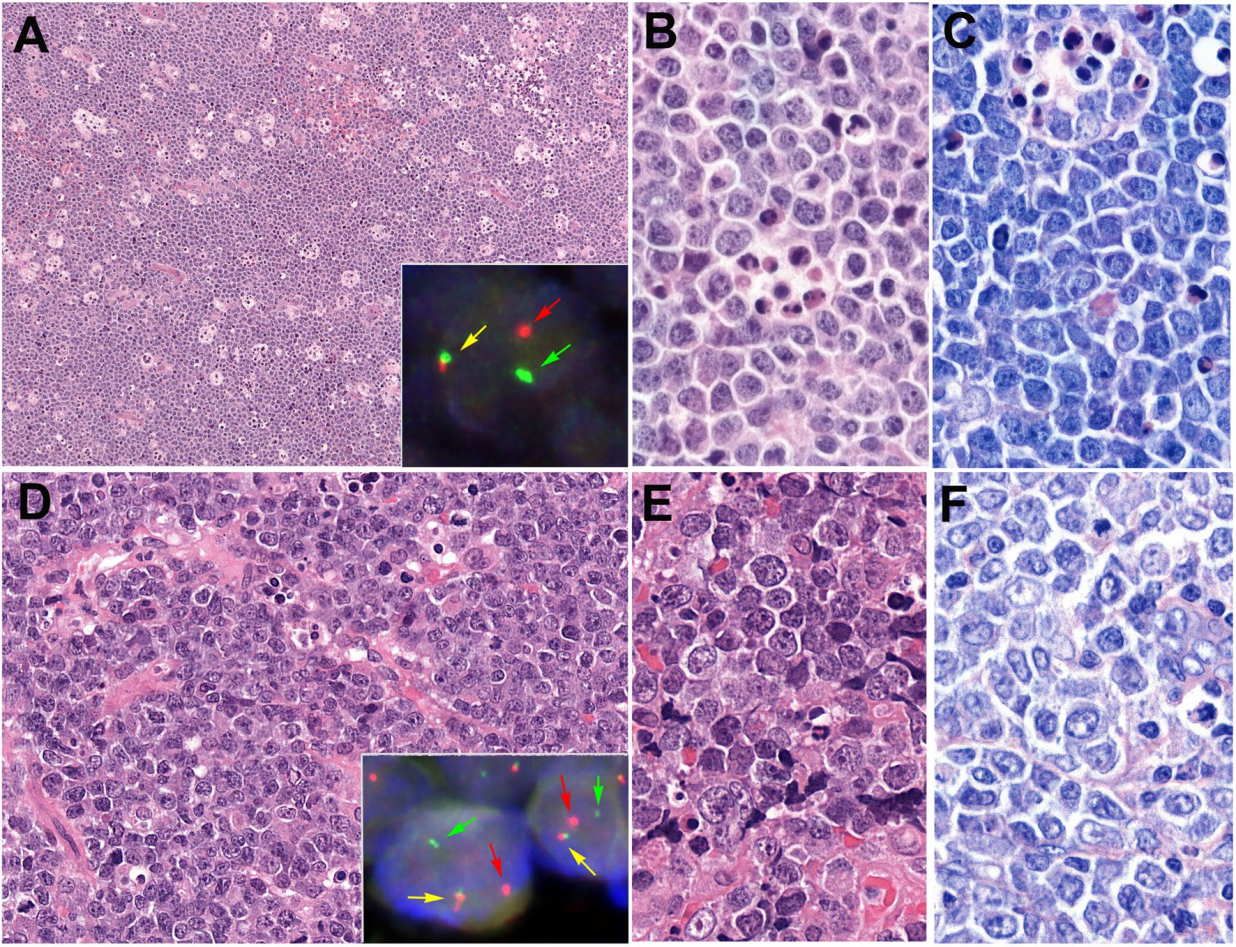
Burkitt lymphoma. **A**, **B**, **C** A case of Burkitt lymphoma with typical morphology with diffuse, monotonous lymphoid infiltration of medium-sized cells with round nuclei, inconspicuous nucleolus, and basophilic cytoplasm highlighted in the Giemsa stain. The cells appear to be cohesive exhibiting squared-off borders (**C**). Note the starry-sky pattern due to the presence of multiple tingible body macrophages with numerous mitoses and apoptotic bodies. Inset: FISH analysis with a *MYC* break apart probe demonstrates a break with one colocalized signal (yellow arrow) and one split signal (green and red arrows) consistent with gene rearrangement (**A**, original magnification, 100 × , hematoxylin and eosin; **B**–**C**, original magnification 630 × hematoxylin and eosin (**B**), Giemsa (**C**)). **D–E** A case of Burkitt lymphoma with large-cell morphologic variation. The cells are rather large, polymorphic, and some have one or several prominent nucleoli. Apoptosis is prominent without a starry-sky pattern. Inset: FISH analysis in two cells with a *MYC* break apart probe demonstrates a break with one colocalized signal (yellow arrow) and one split signal (green and red arrows) consistent with gene rearrangement (**D**, original magnification 200 × ; **E**–**F**, original magnification 630 × , hematoxylin and eosin (**D**–**E**) and Giemsa (**F**))

BL is an aggressive B-cell lymphoma related to the B cells of the dark zone of the germinal center and the most common pediatric NHL. Historically, three clinical variants are recognized: endemic, sporadic, and immunodeficiency-associated. EBV is positive in 100% of the endemic BL but only 30% in the other two clinical variants [44]. In endemic BL, the peak incidence occurs in children (age 6–8 years) with a male predominance and predilection of extranodal sites such as the jaw in younger children or abdomen in older children. Sporadic BL occurs in children, young adults, and elderly patients with male predominance, frequently involves extranodal sites, mostly abdomen (Peyer patches), and associated with EBV infection more commonly in adults than in children. The immunodeficiency-associated BL characteristically presents in the HIV-positive setting with an advanced stage disease and frequent bone marrow and CNS involvement. Diagnostic criteria include morphology (uniform centrally localized medium sized round nuclei, multiple small nucleoli, basophilic vacuolated cytoplasm) and mature B-cell immunophenotype (CD20 + , CD10 + , BCL6 + , BCL2 negative or very weak + , surface immunoglobulin (IG) positive, and TdT negative). A background “starry-sky” pattern is observed with numerous mitoses and apoptotic bodies reflecting the high proliferation rate (Fig. 3A–C). Proliferation should be almost 100% as demonstrated by MIB1 stain. Some morphologic variations (nuclear size, shape, variability) are acceptable in cases with *MYC* rearrangement as this rearrangement is rare in pediatric DLBCL (Fig. 2 D–F). Rare BLs have epithelioid histiocytes and granulomas with a M1/Th1-polarized microenvironment that is associated with favorable prognosis and even with spontaneous remission [51, 52]. Molecular studies showed that rare cases reported as TdT + BL have a molecular signature distinct from BL and are more similar to B-ALL and should be diagnosed as B-ALL with *MYC* rearrangement [53]. Diagnosis of BL requires *MYC* translocation with an IG gene, commonly IGH (80%), followed by IGK (15%), and IGL (5%). The most frequent mutations are in *MYC*, *TCF3*, *ID3*, *SMARCA4*, *ARID1A*, *DDX3X*, and *CCND3* genes [54–56]. Genomic studies have suggested the greater role of EBV in the pathogenesis of BL and have challenged the importance of the historical classification into three groups [56, 57]. Additional studies show that adult and pediatric BL share a common pathobiology with EBV influencing the genetic and molecular subgroups but not entirely depending on EBV status [7, 58]. EBV + BL has more aberrant somatic hypermutations (SHM) but fewer driver mutations. Additionally, the *IG::MYC* rearrangement seems to be the result of SHM, and the tumors carry more often mutations in *DDX3X, GNA13*, and *FOXO1*. In contrast, EBV − BL has more driver mutations and the *IG::MYC* breakpoint is the result of class-switch recombination. These cases carry more mutations in *ID3*, *TCF3*, and *CCND3*. Interestingly, there is a third molecular group with few driver mutations, EBV-positive or -negative characterized by *TP53* mutations. More recently, it was demonstrated that the expression of SOX11 and EBV are mutually exclusive. SOX11 expression occurs in 25–55% of tumors and predominates in pediatric patients [58]. Unlike the adult counterpart, pediatric BL has excellent prognosis with > 90% cure rate [9].

#### High-grade/large B-cell lymphoma with 11q aberration (HG/LBCL-11q)

**Fig. 4:**
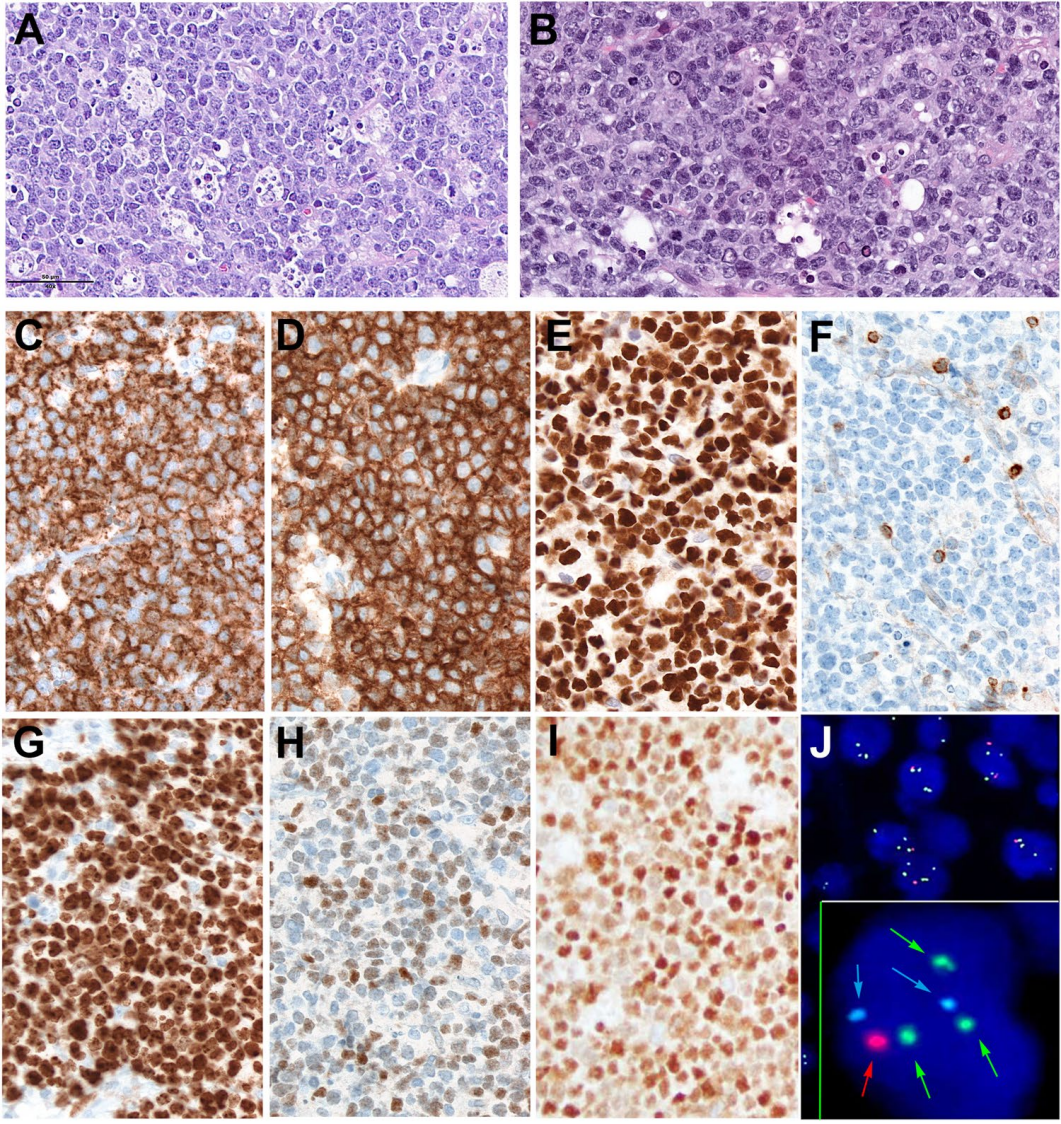
High-grade/large B-cell lymphoma with 11q aberration. **A** H&E stain of case with a monomorphic blastoid morphology mimicking Burkitt lymphoma. The case has a starry-sky pattern with coarse apoptotic bodies. **B** H&E stain of a case showing large-cell morphology. The cells are large with irregular nuclei and prominent nucleoli. There are numerous mitoses and histiocytes but coarse apoptotic bodies are not observed. **C** The tumors cells are CD20, CD10 (**D**), and BCL6 (**E**) positive. **F** BCL2 is negative. Note that the small reactive T cells are positive. **G** The proliferation rate with MIB1 stain is 100%. **H** MYC stain shows heterogeneous and rather weak positivity. **I** LMO2 is positive in the tumor cells. **J** FISH analysis was performed using a commercially available probe. The green arrows show the minimal region of gain in 11q23.3 (spectrum green, Vysis) and the red arrow the loss in 11q24.3 (spectrum red, Vysis. The centromeric probe for chromosome 11 was used (CEP11, spectrum aqua, Vysis, blue arrows) (**A**, **B**, **C**, **D**, **E**, **F**, **G**, **H**, **I**, original magnification, 400 × , hematoxylin and eosin and immunostainings)

HG/LBCL-11q is a mature B-cell lymphoma with a characteristic chromosome 11q-gains/loss pattern and similar morphology/immunophenotype as BL but lacks *MYC* rearrangement or BL-associated molecular mutations [43, 59, 60]. The name high-grade B-cell lymphoma with 11q aberration was chosen in the WHO to stress the high-grade cytology, whereas the ICC preferred the name large B-cell lymphoma with 11q aberration because its biology is closer to DLBCL and very different from BL. The disease predominates in children and can occur in the setting of immunodeficiency, including HIV, posttransplant, and ataxia-telangiectasia. HG/LBCL-11q is predominantly a nodal disease presenting most commonly in head and neck lymph nodes (60–70%) but also in extranodal sites (gastrointestinal tract, in 30–40% of the cases). Although most cases have blastoid morphology, some cases have an intermediate or large cell morphology (Fig. 4) [43]. The starry-sky pattern with coarse apoptotic bodies, observed in 50% of the cases, suggests the diagnosis of HG/LBCL-11q [43]. While the immunophenotype is similar to BL, it differs with frequent expression of LMO2, CD16, and CD56 [61]. The defining genetic event is a complex aberration involving the long arm of chromosome 11q, showing a gain in 11q23.2–23.3 and a telomeric loss in 11q24.1-qter, best detected by high-resolution array-based genomic hybridization, in the absence of *MYC*-R [62]. FISH analysis with commercially available probes, although not as sensitive, is acceptable for diagnosis. [43] The presence of gains in 11q23.3 alone is considered non-specific. In contrast, the telomeric loss alone of 11q24.1 is considered specific for this disease and enough to render the diagnosis of HG/LBCL-11q. The demonstration of *MYC* translocation in some cases is controversial; however, until more studies are available, the presence of *MYC* translocation should be an exclusion criteria for the diagnosis of HG/LBCL-11q. The molecular mutational profile of HG/LBCL-11q is closer to DLBCL of GCB-type than to BL, and unlike BL it has frequent *GNA13* mutations (50% of cases). The prognosis is better than BL with 100% overall survival (OS) [23, 59, 60].

### Mature B-cell lymphoma with small B-cell morphology

Small B-cell lymphomas are rare in children. The most frequent indolent lymphoma in the pediatric age is pediatric-type follicular lymphoma (PTFL) that differs clinically, morphologically, and genetically from the adult counterpart. Pediatric nodal marginal zone lymphoma (PNMZL) and PTFL represent overlapping entities with marked clinical and morphological similarities. Current WHO classification considers these two separate entities, but recent studies recommend merging these two entities under the name of PTFL with and without marginal zone differentiation, and therefore, will be discussed together in this review [63]. Chronic lymphocytic leukemia (CLL), although rare, may occur in children and young adults and are morphologically and genetically similar to the adult counterpart [64].

#### Pediatric-type follicular lymphoma (PTFL) and pediatric nodal marginal zone lymphoma (PNMZL)

**Fig. 5:**
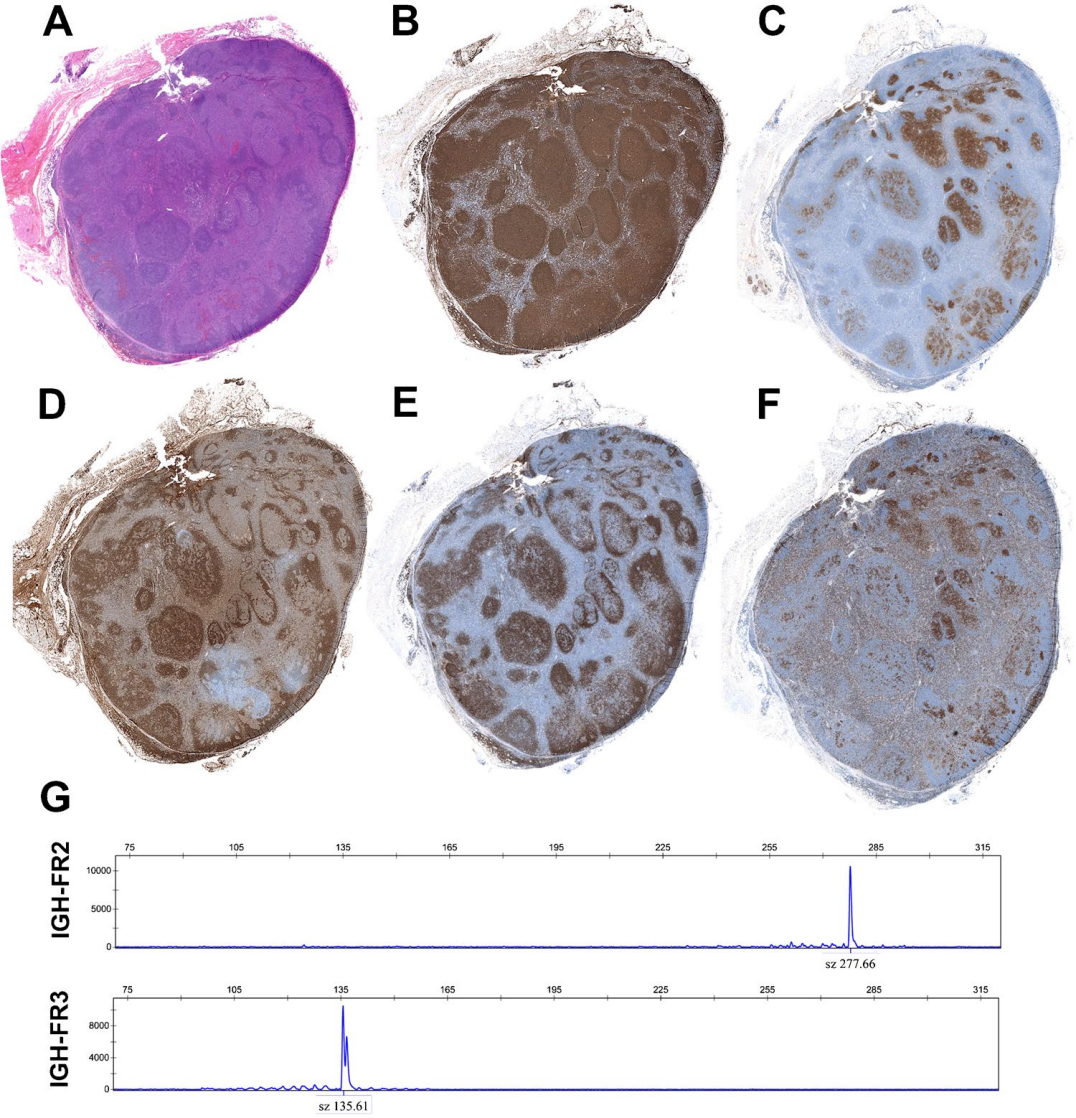
Pediatric-type follicular lymphoma with marginal zone differentiation.** A** Panoramic view of a lymph node with large irregular follicles and attenuated mantle zones on the right side, whereas the left side shows follicles with PTGC like changes. **B** CD20 stain reveals the nodular growth pattern with few spill-over into the interfollicular areas. **C** CD10 is positive in the germinal center cells. Note the difference in distribution of the stain in the left and right regions of the lymph node. **D** IgD stain highlights the PTGC-like features on the left side and the expanded follicles with attenuated mantle zones on the right side. **E** CD23 stain is similar to IgD. **F** MIB1 stain is similar to CD10. Note the high proliferation in the expanded follicles on the right and the residual germinal center cells on the left (**A**, **B**, **C**, **D**, **E**, **F** snap-shots). **G** IGH clonality analysis using Biomed-2 primers. FR2 and FR3 reveal a monoclonal peak at 277 and 135 bp, respectively

PTFL is rare (< 2% of pediatric NHL) and characterized by a neoplastic proliferation of follicular germinal center B cells. PTFL predominantly affects head and neck lymph nodes of male patients (M:F; 20:1), who present with limited clinical stage (mostly stage I disease) and has an invariably indolent behavior permitting a conservative watch and wait approach after complete excision of the involved lymph node. Ocular/conjunctival presentation is rare but well documented [65]. Morphologically, it is characterized by expansile, serpiginous, and sometimes confluent follicles with attenuated mantle zones (Fig. 5). The follicles are composed of medium-sized to large blastoid cells, but in some cases centroblasts predominate with a starry-sky pattern but without polarization, a feature that might help in the differential diagnosis with reactive germinal centers. In many instances, a rim of normal-appearing nodal tissue is observed giving the impression of a “node within a node.” The tumor cells are positive for germinal center markers (CD10 and BCL6) but remain negative for BCL2 and MUM1/IRF4. The demonstration of a monoclonal B-cell population is required for the diagnosis to exclude florid hyperplasia. In previous studies, it has been reported that some PTFL display marginal zone differentiation (CD20 in the interfollicular areas and progressive transformation of germinal centers (PTGC)-like features highlighted with IgD and CD23 stain), and some PNMZL have germinal center markers (CD10 expression often demonstrated by flow analysis), suggesting that PTFL and PNMZL may represent a morphological spectrum within the same biological entity (Fig. 5) [63]. These two disorders not only share the indolent clinical behavior and similar morphology but they also share the genetic features. Both disorders have low genomic complexity and the same mutational profile, including mutations in *TNFRSF14*, *MAP2K1*, and *IRF8* [66–70]. The disease lacks rearrangement in *BCL2*, *BCL6*, I*GH*, *MYC*, and *IRF4* loci [64, 66]. Prognosis is excellent with > 95% 5-year OS [6, 64, 66, 71].

### Precursor T/NK-cell lymphomas

#### T- and NK-lymphoblastic lymphoma

T-lymphoblastic lymphoma (T-LBL) is a common pediatric neoplasm (30–40% of pediatric NHL) of precursor T cells that commonly presents as a mediastinal mass with variable BM and PB involvement (by definition < 25% BM or PB blasts). Diagnostic criteria are morphologic presence of blasts with T cell (cytoplasmic or surface CD3) and immature (CD34, CD117, TdT, CD1a) immunophenotype, ideally distinct from normal thymic lymphocytes or evidence of PB or BM involvement. Rare cases of early T-precursor (ETP) subtype of T-LBL are reported [72]. Less commonly, precursor T-cell neoplasms can present as leukemia (T-ALL) with ≥ 25% BM or PB blasts or ≥ 20% blasts without mass. The same molecular mutations are seen in both pediatric T-LBL vs. T-ALL, but they vary in frequency [73]. As with B-LBL, the outcome for T-LBL with current therapy is excellent with > 90% 3-year EFS [20].

NK-lymphoblastic lymphoma is defined as a neoplasm of precursor of NK cells and is included as a provisional entity in the 2017 WHO classification. Although, listed as an entity in the pediatric WHO classification, there are no robust criteria for its diagnosis or for its distinction from blastic plasmacytoid dendritic cell neoplasm and myeloid sarcoma (acute undifferentiated leukemia or minimally differentiated AML). This subtype remains a provisional entity in ICC but removed, for now, in the current WHO 5th classification scheme.

### Mature T/NK-cell neoplasms

Pediatric mature T/NK-cell neoplasms are rare (< 2% of pediatric NHL). Unlike B-cell lymphoma where the cytology of the tumor cells aids in subclassification, the cytology of all mature T/NK-cell neoplasms can vary from small benign-appearing lymphocytes to large anaplastic-transformed cells. Clinical history (particularly for cutaneous lesions), immunophenotyping, and molecular findings are critical for appropriate subclassification. Peripheral T-cell lymphomas (PTCL), in general, are rare tumors in children, adolescents, and young adults with poor prognosis and scarce genetic data [12].

#### Peripheral T-cell lymphoma, not otherwise specified (PTCL, NOS)

PTCL, NOS is defined as mature T-cell lymphoma that does not meet criteria for another specific T-cell lymphoma subtype. Diagnostic criteria are effacement of normal nodal architecture by clonal T cells and exclusion of a specific T-cell lymphoma subtype. PTCL, NOS is the second most frequent mature T-cell lymphoma in children after anaplastic large-cell lymphoma, ALK positive (ALCL, ALK +). Molecular studies show that the mutational profiles in children and adults differ with low frequency of mutations observed in T-follicular helper (TFH) cell lymphomas (*IDH2, RHOA, TET2*), and lower frequency of *TP53* mutations [11, 12]. Accordingly, TFH lymphoma practically does not exist in children, and therefore, was not included in the pediatric lymphoma classification. Overall prognosis is poor with 56–61% 5-year EFS [9, 74].

#### Aggressive NK-cell leukemia (ANKL)

ANKL is rare, most prevalent in Asia, and is defined as a systemic proliferation of neoplastic NK cells involving multiple organs (most commonly BM, PB, and spleen). Patients present acutely with fever, cytopenia, liver failure, coagulopathy, and hemophagocytic lymphohistiocytosis (HLH). EBER is positive in 100% of the pediatric cases; however, in adults (mainly in elderly patients), approximately 10% of the cases are EBV negative [75]. Cytology varies from mature large granular lymphocytes to enlarged more pleomorphic forms. Diagnostic criteria are acute presentation, fever, and systemic proliferation of NK cells (CD2 + , sCD3 − , CD3ε + , CD5 − , CD56 + , and positivity for cytotoxic markers) with absent T-cell receptor (TR) expression or gene rearrangement [76]. Rare cases are believed to arise from chronic active EBV disease, systemic, extranodal NK/T-cell lymphoma, or NK-large granular lymphocyte (LGL) leukemia. Complex karyotype with unbalanced chromosomal abnormalities as well as non-specific recurrent alterations have been identified. Importantly, the clinical presentation, morphology, and molecular features are similar to systemic EBV + T-cell lymphoma of childhood (SEBVTCL). The difference is the EBV infected cell; ANKL is of NK-cell derivation and SEBVTCL of T-cell derivation. Prognosis is dismal with less than 2 months OS [77, 78].

#### Subcutaneous panniculitis-like T-cell lymphoma (SPTCL)

SPTCL is a rare PTCL representing 0.1–0.25% of pediatric NHL [79]. Patients present with multiple subcutaneous lesions and may have systemic symptoms. Diagnostic criteria consist of a lobular subcutaneous infiltrate, rimming of adipocytes by neoplastic lymphocytes, fat necrosis, and karyorrhectic debris. The neoplastic cells are CD3 + , CD8 + , cytotoxic granules + , and TCRαβ+ but negative for CD4, CD56, and EBER. CD5 is usually negative or weak. Proliferation is high. A clonal T-cell rearrangement is often present [80–82]. Recently, germline mutations of the *HAVCR2* gene have been demonstrated [83]. Two hot-spot mutations have been identified, one predominates in Europeans (*HAVCR2*^I97M^) and the other in East Asia (*HAVCR2*^Y82C^). *HAVCR2* encodes for TIM3, a protein involved in regulating T-cell inflammation. Patients with *HAVCR2* mutations are younger (< 30 years), often associated with HLH and worse prognosis (5-year OS 45%) [84]. SPTCL in children has a good prognosis with 85% 5-year OS [80–82, 85, 86].

#### Hepatosplenic T-cell lymphoma (HSTL)

HSTL is a rare pediatric T-cell lymphoma that infiltrates the sinusoids of the liver, spleen, and BM but spares LN [79]. Approximately 40% of the cases occur in the setting of immune suppression, particularly in solid organ transplant recipients or patients with Crohn’s disease treated with anti-tumor necrosis factor drugs or thiopurines. Patients present with hepatosplenomegaly, B symptoms, and cytopenia, especially thrombocytopenia. The cytology of the tumor cells is variable (medium-sized, large-sized, pleomorphic, blastic) with sinusoidal distribution in the spleen, liver, and BM [87]. Diagnostic criteria are the above morphologic findings with cytotoxic T-cell immunophenotype that usually express γδ TCR, occasionally αβ TCR, or rarely (< 5%) TCR-silent. The cells have a non-activated cytotoxic phenotype demonstrated by positive TIA1 expression but negative for granzyme B and perforin. The tumor cells are CD2 + , CD3 + , CD7 ± , CD56 ± , CD5 − , and CD30 − and usually CD4/CD8 double negative with rare cases being CD8 + . Chromosomal alterations include isochromosome 7q (80%), trisomy 8 (50%), and mutations include alterations in *SETD2*, *INO80*, *TET3*, *SMARCA2*, *STAT5B*, *STAT3*, *TP53*, and *IDH2* [57, 88]. Prognosis is worse than PTCL, NOS with 17% 5-year EFS [74].

#### Anaplastic large-cell lymphoma (ALCL)

Systemic ALCL can be divided in two groups based on the presence or absence of *ALK* rearrangement on chromosome 2p23. The most frequent partner gene is *NPM1* on chromosome 5q35.1; however, many fusion partners have been described, all leading to expression of oncogenic ALK fusion protein.

#### Anaplastic large-cell lymphoma, ALK positive

ALCL, ALK + is a common mature T-cell lymphoma and represents 15% of pediatric NHL. It is the most frequent mature T-cell lymphoma in children. Morphologically, ALCL is composed of large anaplastic cells with abundant cytoplasm, horseshoe-shaped nucleus, and a perinuclear eosinophilic region that are localized in the sinusoidal space of lymph nodes in cohesive groups raising the differential diagnosis with metastatic carcinoma. Several histologic variants are recognized, including the common variant (60%), the lymphohistiocytic variant (10%), the small-cell variant (5–10%), the Hodgkin-like variant (3%), and cases with more than one pattern. Diagnostic criteria are uniform positivity for CD30 + with ALK1 expression. The cells express T-cell markers (CD2, CD3, CD5, CD7, CD43), TIA1, granzyme B, perforin, and EMA; although, some cases have variable loss of these markers. The most reliable T-cell marker for the diagnosis of ALCL is CD2. Molecular studies show consistent *ALK1* rearrangement with multiple partners without prognostic differences. More recently, studies showed additional molecular heterogeneity with some associated with increased chance of relapse [89]. The prognosis of pediatric ALCL, ALK + (65% to 85% 5-year OS) is similar to that in the adult counterpart and is worse than pediatric B-cell NHLs [9, 90]; the morphologic variant with small-sized tumor cells and/or lymphohistocytic background as well as minimal disseminated diseases in BM or PB are associated with increased treatment failure/relapse [91].

#### Anaplastic large cell-lymphoma, ALK negative

Pediatric ALCL, *ALK − *counterpart is rare and little is known about its characteristics, unlike the more common adult counterpart with identified genetic and molecular mutations [92, 93] and worse prognosis. ALCL, *ALK − *by definition, lacks *ALK* rearrangement, and although it is considered a single disease, novel molecular studies in adult cases indicate the existence of several distinct genetic subgroups [92]. The relevance of these genetic subgroups in pediatric cases is still to be demonstrated [57, 74, 79, 87, 88].

### EBV-positive lymphoproliferative diseases of childhood

EBV + T and NK lymphoproliferative disorders (LPD) in childhood are uncommon disorders that affect mainly pediatric and young adult populations but can also occur in adults. This group of diseases are more frequent in Asian and Latin American populations. The reviewed classification recognizes four major disorders: hydroa vacciniforme (HV) LPD, severe mosquito bite allergy, systemic chronic EBV (CAEBV) disease, and SEBVTCL. The criteria and morphological features of these four diseases have been recently reviewed [94].

#### Systemic EBV-positive T-cell lymphoma of childhood

**Fig. 6:**
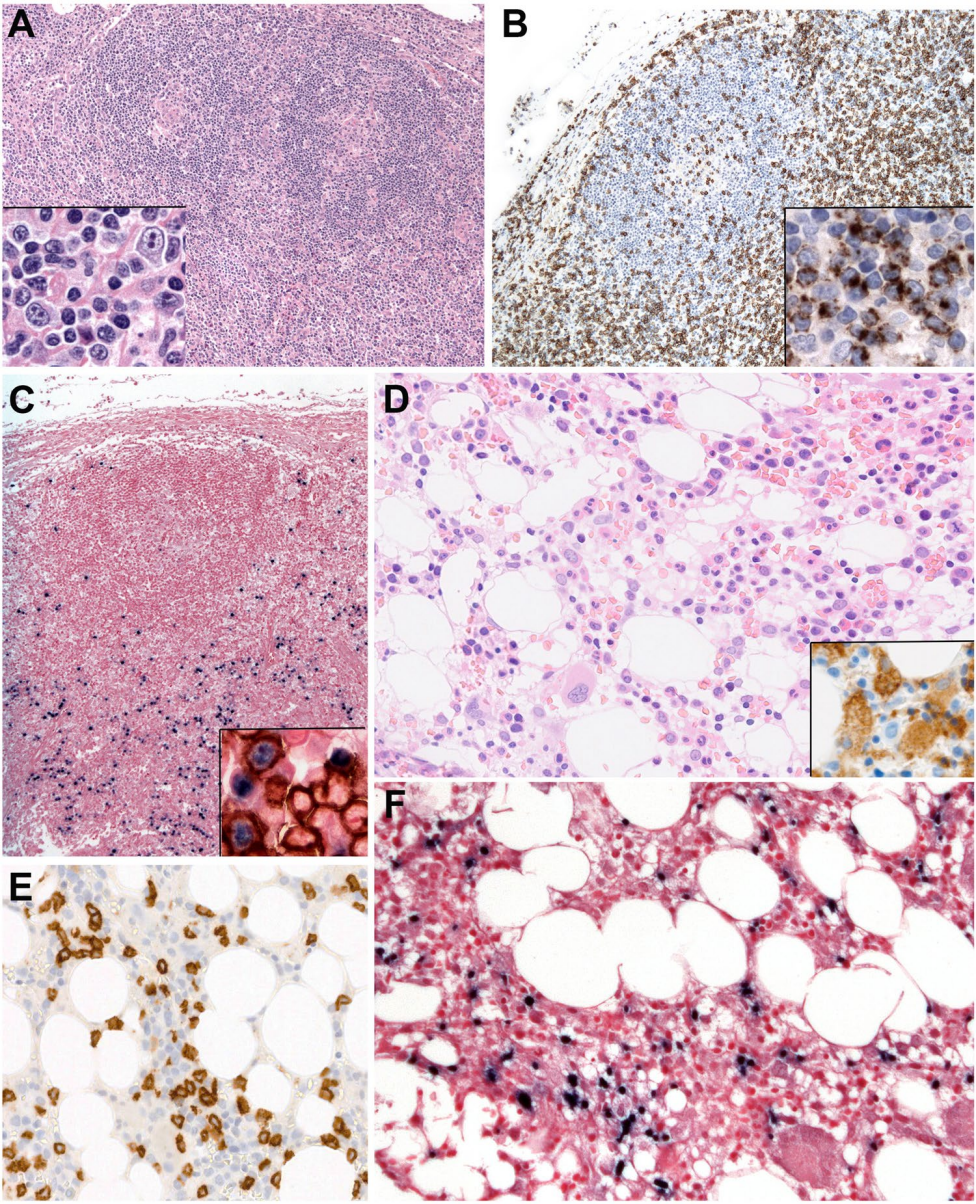
Systemic EBV + T-cell lymphoma of childhood. **A** Lymph node with residual regressive germinal centers and expanded interfollicular region. Inset: the interfollicular infiltrate is polymorphic with small cells and some relatively large cells with prominent nucleoli and abundant cytoplasm. **B** The infiltrate in the interfollicular areas is predominantly CD8 positive. Inset: the cells express TIA1. **C** Many cells are positive for EBV-encoded small RNA (EBER). Inset: double staining demonstrates that the EBER positive cells (nuclear black staining) are CD8 positive (membranous brown staining). Note that only a minority of the CD8 positive cells are EBER positive. **D** The bone marrow is relatively hypocellular and shows histiocytic hyperplasia with erythrophagocytosis. Inset: CD163 highlights the presence of abundant histiocytes with erythrophagocytosis. **E** CD8 + T cells infiltrate the bone marrow. **F** The infiltrating T cells are EBER positive in the bone marrow

SEBVTCL is a rare but life-threatening disease of children and adolescence characterized by a clonal proliferation of EBV-positive T cells, mostly CD8 + with an activated cytotoxic phenotype (TIA1 + , granzyme B + , perforin +) [95, 96]. The disease typically occurs shortly after primary acute EBV infection and rarely in the setting of CAEBV disease. Patients present with fever, general malaise, upper respiratory symptoms, pancytopenia, hepatosplenomegaly, liver failure, and neurological symptoms within a period of weeks to months. The disease is for the most part complicated by HLH, coagulopathy, sepsis, multi-organ failure, and finally death within days to weeks of the initial diagnosis. Some children present mainly with lymphadenopathy but with the same fulminant clinical course. The EBV serology is often negative for anti-VCA IGM, a feature that is misleading and delays the diagnosis since acute EBV infection is not considered. In contrast, EBV DNA copy numbers in blood are very elevated and help to confirm the diagnosis. The diagnosis is rendered either in a BM or liver biopsy, and rarely in a lymph node biopsy (Fig. 6) [95, 97]. The cytology of the cells is variable ranging from benign small mature lymphocytes to pleomorphic medium- to large-sized cells associated with striking hemophagocytosis. The infiltrating cells are CD8 + , CD3 + , CD2 + , CD56 − , cytotoxic, and αβ T cells. Rare cases are CD4 + or with CD4/CD8 co-expression. In difficult cases, double staining with EBER/CD4 and EBER/CD8 is useful to determine the lineage of the infected cells. Diagnostic criteria include the demonstration of a clonal EBV + T-cell population. Prognosis is poor with most cases resulting in death within weeks of diagnosis [98, 99]. The only treatment known is bone marrow transplantation (BMT).

#### Hydroa vacciniforme lymphoproliferative disorder

HV-LPD is a rare cutaneous EBV-driven T-cell proliferative disorder with potential progression to systemic lymphoma [94, 100, 101]. Two clinical forms are recognized: classic and systemic. The classic form is an indolent disease, self-limited in the adolescence, characterized by papulo-vesicular eruptions on sun-exposed skin areas, and no systemic symptoms. The classic form is associated with increased numbers of gamma delta T cells in the PB and in the skin. There is a seasonal variation with increased recurrence in spring and summer and good response to photoprotection. Rarely, patients progress to a more aggressive (systemic) form. The systemic form commonly affects Asians and Latin American populations. The skin lesions present in sun exposed and non-exposed areas and are accompanied by systemic symptoms including fever, wasting, lymphadenopathy, and hepatosplenomegaly. The disease follows a protracted course and patients may have recurrent skin lesions for as long as 10–15 years before developing a more aggressive disease [102]. Patients might respond initially to immunomodulating therapies but eventually BMT is needed [103]. Diagnostic criteria for both forms require appropriate clinical findings combined with typical morphologic findings of intraepidermal spongiotic vesiculation, perivascular, and periadnexal lymphocytic infiltrate containing scattered EBER-positive cytotoxic T or NK cells [104]. The infiltrating cells are mostly CD8 + with few cases being CD4 + or double CD4/CD8 + . NK phenotype also occurs, and these cases tend to be more panniculitic and might mimic SPTCL. CD30 is often expressed in variable proportion or tumor cells. LMP1 is usually negative. Most cases have a clonal T-cell population but its presence does not correlate with an aggressive behavior. EBV DNA is elevated in blood and the levels do not discriminate between the classic and systemic form of the disease [100, 101].

### Hodgkin lymphoma

Hodgkin lymphoma is a frequent lymphoma in children and young adults. It represents 8% of pediatric cancer similar to that of pediatric NHL; however, it represents 46% of all lymphomas in pediatric age. The classification and diagnostic criteria are the same as in the adult counterpart. The subclassification of Hodgkin lymphoma into classic Hodgkin lymphoma (CHL) and nodular lymphocyte predominant Hodgkin lymphoma (NLPHL) remains unaltered. However, the ICC has proposed to rename NLPHL as nodular lymphocyte predominant B-cell lymphoma (NLPBL), and to reserve the “Hodgkin lymphoma” name only for the classic form [15, 105]. Recent studies have stressed the important role of tumor microenvironment and new immunohistochemical markers in the pathogenesis and diagnosis of CHL.

#### Classic Hodgkin lymphoma

CHL represents 90–95% of pediatric HL and is primarily a nodal disease with frequent involvement of the mediastinum. Cases resembling CHL but presenting primarily in extranodal sites probably represent a different disease. CHL has four morphological subtypes: nodular sclerosis (NS), mixed cellularity (MC), lymphocyte-rich (LR), and lymphocyte-depleted (LD), largely based on the background inflammatory cell types and fibrosis. NS subtype is more prevalent in the developed countries while MC subtype is more prevalent in the developing countries. This sub-classification is irrelevant for therapeutic decisions and prognosis but important in the initial differential diagnosis and workup. CHL is characterized by scattered large atypical defective B cells, which are mono-, bi-, or multi-nucleated with prominent nucleoli, and abundant cytoplasm known as Reed-Sternberg (RS) cells, within a background of small T or B lymphocytes, eosinophils, histiocytes, plasma cells, and fibrosis. Diagnostic criteria are these morphologic findings with defective B-cell immunophenotype of the large, atypical cells (CD30 + , PAX5dim + , CD20 ± , CD79b ± , CD45 − ; ideally CD15 + , OCT2 − , and/or BOB1 − , MEF2B − , and BCL6 −) [106]. RS cells are also positive for MUM1, GATA3, and IMP3 [105]. In Western countries, up to 25% of CHL are EBV-positive; however, in developing countries and pediatric populations, the association is much higher [107]. EBV detection in the RS cells by LMP1 or EBER in situ hybridization is recommended because when present aids in the diagnosis and suggests possible biology. Molecular studies on isolated RS cells show higher mutation burden in pediatric HL compared to adult HL [108]. Prognosis is excellent with 96% 5-year OS [10] and significantly better than the 88% seen in the adult counterparts.

#### Nodular lymphocyte-predominant Hodgkin lymphoma

NLPHL represents 5–10% of pediatric HL and is characterized by scattered large, atypical B cells with multi-lobated nuclei, small to indistinct nuclei (popcorn cells, lymphocyte-predominant/LP cells) within a background of mostly small B- and T-lymphocytes with variable histiocytes and dendritic cells. Most patients are male and present with isolated lymphadenopathy without B symptoms, and low-stage disease. Morphologically, the composition of the background varies and six patterns are identified, A to F, so-called “Fan patterns” [109]; some associated with good (A, B) and some with worse prognosis and more frequent recurrences (D, E, F). The prognostic significance of pattern C is controversial but seems to be of favorable prognosis. Diagnostic criteria consist of the above morphology and B-cell immunophenotype of the large, atypical cells (positive for CD19, CD20, CD79a, PAX5, CD45, BCL6, and MEF2B) that are rosetted by CD3 + TFH cells expressing CD4, PD1, CD57, and ICOS [106]. In cases with patterns D–F, there is a progressive loss of the characteristic nodular pattern and there is a reduction of reactive B cells, loss of residual follicular dendritic cells, and increased infiltration of T cells and histiocytes showing overlap with de novo T-cell/histiocyte-rich large B-cell lymphoma. Occasional to rare cases have abnormal expression of CD30, CD15, and/or EBER that are more frequently associated with CHL [110, 111]. A subset of cases usually in young men with cervical lymphadenopathy and diffuse pattern, express IgD and can be associated with *Moraxella catarrhalis* suggesting an antigen-driven pathogenesis [112, 113]. The expression of IgD and MEF2B in the tumor cells is very helpful to confirm the diagnosis of NLPHL vs. CHL, especially in needle biopsies [113]. Despite increased recurrence with some histologic subtypes, the prognosis is excellent (> 95% 5-year OS) and unlike adults, curative surgical excision is possible in localized disease.

## Conclusion and future directions

We provide an overview of some pediatric lymphomas recognized by the new Pediatric WHO classification as well as some pediatric subtypes that have yet to be recognized. Although the diagnostic criteria for adult and pediatric are similar, there are differences in relative frequency and genetic background with the pediatric counterpart often having better prognosis. The index case is PTFL, initially identified in children and later in adults, with absent *IGH::BCL2* and better prognosis compared to follicular lymphoma. Future studies will eventually determine if other pediatric subtypes with better prognosis can also occur in adults, leading to better prediction of outcome and understanding of the underlying mechanism.
